# Characterization of Anti-Angiogenic Chemo-Sensitization via Longitudinal Ultrasound Localization Microscopy in Colorectal Carcinoma Tumor Xenografts

**DOI:** 10.1109/TBME.2021.3119280

**Published:** 2022-03-18

**Authors:** Matthew R. Lowerison, Wei Zhang, Xi Chen, Timothy M. Fan, Pengfei Song

**Affiliations:** Beckman Institute, University of Illinois at Urbana–Champaign, USA; Department of Electrical and Computer Engineering, University of Illinois at Urbana–Champaign, USA.; Beckman Institute, University of Illinois at Urbana–Champaign, USA; Department of Electrical and Computer Engineering, University of Illinois at Urbana–Champaign, USA; Department of Medical Ultrasound, Tongji Hospital,Tongji Medical College, Huazhong University of Science and Technology, China.; Beckman Institute, University of Illinois at Urbana–Champaign, USA; Department of Electrical and Computer Engineering, University of Illinois at Urbana–Champaign, USA.; Department of Veterinary Clinical Medicine, University of Illinois Urbana–Champaign, USA; Cancer Center at Illinois, University of Illinois Urbana–Champaign, USA.; Beckman Institute, University of Illinois at Urbana–Champaign, Champaign, IL 61801 USA; Department of Electrical and Computer Engineering, University of Illinois at Urbana–Champaign, Champaign, IL 61801 USA; Cancer Center at Illinois, University of Illinois Urbana-Champaign, Champaign, IL USA.

**Keywords:** Cancer, colorectal carcinoma, contrast agents, microbubbles, microvessels, super-resolution, therapy, ultrasound localization microscopy

## Abstract

**Objective:**

Super-resolution ultrasound localization microscopy (ULM) has unprecedented vascular resolution at clinically relevant imaging penetration depths. This technology can potentially screen for the transient microvascular changes that are thought to be critical to the synergistic effect(s) of combined chemotherapy-antiangiogenic agent regimens for cancer.

**Methods:**

In this paper, we apply this technology to a high-throughput colorectal carcinoma xenograft model treated with either the antiangiogenic agent sorafenib, FOLFOX-6 chemotherapy, a combination of the two treatments, or vehicle control.

**Results:**

Longitudinal ULM demonstrated morphological changes in the antiangiogenic treated cohorts, and evidence of vascular disruption caused by chemotherapy. Gold-standard histological measurements revealed reduced levels of hypoxia in the sorafenib treated cohort for both of the human cell lines tested (HCT-116 and HT-29). Therapy resistance was associated with an increase in tumor vascular fractal dimension as measured by a box-counting technique on ULM images.

**Conclusion:**

These results imply that the morphological changes evident on ULM signify a functional change in the tumor microvasculature, which may be indicative of chemo-sensitivity.

**Significance:**

ULM provides additional utility for tumor therapy response evaluation by offering a myriad of morphological and functional quantitative indices for gauging treatment effect(s).

## I. Introduction

METASTATIC colorectal cancer (mCRC) is the third leading cause of cancer-related mortality in the United States [1]. Systemic chemotherapy combined with anti-angiogenic (AA) agents, specifically Bevacizumab, plays a substantial role in the clinical treatment regimen of this patient population; it is the first-line neoadjuvant therapy used to clinically downstage mCRC tumors prior to surgery and it is also the first-line treatment option for patients with advanced-stage mCRC who are not amenable to surgical resection [2]. Several phase II and III clinical trials have demonstrated an improvement in patient outcome and longer, progression-free survival for mCRC patients treated with a combination AA/chemotherapy regimen versus chemotherapy alone [3-6]. The addition of AA agents is thought to be essential to the efficacy of this combined therapy in part via the phenomenon of vascular normalization [7], where the selective remodeling of chaotic intratumoral vasculature can alleviate interstitial hypertension, improving blood flow and the delivery of chemotherapeutics inside the tumor [8] while simultaneously relieving a hypoxic tumor microenvironment known to promote chemotherapy resistance [9]. However, combination therapies are associated with decreased treatment tolerability and increased side effects and adverse events, leading to a higher incidence of treatment discontinuation and dose reduction that may obscure a synergistic benefit [10]. Accurate predictors of biologic response to AA therapy are therefore of vital clinical significance to mCRC as they permit the early identification of non-responders, sparing patients from unnecessary toxicity and medical expenses, and can potentially allow for individualized treatment strategies to optimize therapeutic stratification by serially identifying and measuring patient responses before a survival benefit is observed.

However, there is currently no validated predictive biomarker for AA response in mCRC [11], invariably leading to the treatment of molecularly unselected patient populations, and a reliable clinical assessment tool for AA therapies does not currently exist. Conventionally, radiographic assessment of tumor treatment response depends on longitudinal changes in tumor burden and size, such as the RECIST criteria [12] or the WHO criteria [13]. These criteria are well suited to cytoreductive regimens, such as chemotherapy and radiation therapy, but are poorly indicative of anti-tumoral responses to molecular-targeted therapies such as AA agents. In addition, these criteria are insensitive to the intratumoral vascular changes that are thought to be critical to the synergistic effect(s) of combined chemotherapy/AA regimens. The limitations of objective response criteria in evaluating AA mono- and combined-therapies is well established in literature [14], [15] and has motivated the development of a wide-spectrum of surrogate “imaging biomarkers” [16], [17] for the purposes of detecting early tumor response(s) to AA therapies. In particular, functional imaging modalities, such as dynamic contrast-enhanced magnetic resonance imaging (DCE-MRI), dynamic contrast-enhanced X-ray computed tomography (DCE-CT), fluorodeoxyglucose-positron emission tomography (FDG-PET), and contrast-enhanced ultrasound (CEUS) have all shown ample evidence for satisfying this gap in the clinical management armamentarium [16-18]. However, these modalities are incapable of resolving vasculature features down to the capillary scale and must instead infer microvascular changes based on indicator-dilution analyses. The quantifications derived from these modalities are dependent on the selected pharmacokinetic model and require strict imaging normalization and standardization protocols that may be impractical for widespread clinical implementation.

A recently proposed super-resolution ultrasound-based vascular imaging modality offers a potential solution. Ultrasound Localization Microscopy (ULM) [19-21] leverages clinically used contrast-enhancing microbubbles (MB) with state-of-the-art ultrasound technologies to improve imaging resolution by a factor of ten [22] while maintaining the imaging penetration depth, resulting in clinically relevant microvascular network reconstructions that can span an entire organ. ULM is of great interest for tumor AA response evaluation given that it resolves the trajectory and velocity of a purely intravascular contrast agent, potentially down to the microvascular scale, providing quantitative indices of intratumoral vascular supply while avoiding the confounding effects of vascular permeability. The technique also shares the high safety profile of clinically used CEUS, with minimal invasiveness and no ionizing radiation, permitting rapid and agile longitudinal study designs. It is therefore not surprising that ULM has been previously applied to tumor vascular characterization. Ackermann and Schmitz [23] reconstructed the microvessels of a mouse tumor xenograft using ULM. Opacic *et al*. [24] produced a tumor classifier for mouse tumor xenografts based on the morphological and physiological parameters derived from their motion-model ULM technique. Dencks *et al*. [25] then applied this model to a clinical pilot study on triple-negative breast cancer patients treated with neoadjuvant chemotherapy. Lin *et al*. [26] used ULM on a rat model of fibrosarcoma and found a significant increase in the vascular tortuosity of tumors compared to normal tissue, as measured by the distance metric (DM) [27]. Our group [28] found that increased DM in ULM images was significantly correlated with a gold-standard measurement of hypoxia in a renal cell carcinoma tumor xenograft in the chorioallantoic membrane (CAM) of chicken embryos. Furthermore, a related field of tumor characterization has focused on the description of tumor vasculature in terms of its fractal dimension [29], [30], but this remains an unexplored index in ULM imaging. Gazit *et al*. [30] demonstrated that human CRC xenografts in a murine dorsal chamber preparation will start with a fractal dimension around 1.7, which is consistent with diffusion-limited structures embedded in two dimensions [31] but will rapidly plateau to a steady state of 1.84 due to heterogenous local pro-angiogenic substrates arising from cancerous growth. During tumor regression, Gazit *et al*. demonstrated that the fractal dimension of pathological vasculature will begin to reduce back to a level between that of healthy tissue and growing tumor. Given that ULM can provide high-fidelity microvascular structural and velocity information throughout an entire tumor mass and can generate surrogate imaging biomarkers correlated to intratumoral hypoxia, we posit that this technique can be used to explore the chemo-sensitizing effect of AA agents.

In this paper, we present the results of ULM imaging applied to a combined chemotherapy/AA regimen on a human CRC tumor xenograft model engrafted into the CAM of chicken embryos. HCT-116 (ATCC CCL-247) and HT-29 (ATCC HTB-38) tumor xenografts were treated with either sorafenib, FOLFOX-6, a combination of the two therapies, or vehicle control. Contrast-enhanced ultrasound in-phase/quadrature (IQ) demodulated data were acquired from 63 CAM tumors using a Verasonics Vantage 256 system with a L35-16vX linear array transducer both before and after therapy. Super-resolution ULM images (reconstructed at a 5 μm axial/lateral resolution) were analyzed and compared against gold-standard histological quantifications of cell proliferation and hypoxia.

The rest of the paper is structured as follows. In the Materials and Methods section, we introduce the chicken embryo tumor xenograft model of human CRC, describe the preparation and dosing of AA and chemotherapeutic agents, and describe the microbubble injection, ultrasound imaging acquisition, and super-resolution processing techniques used for ULM of this tumor model. In the Results section, we demonstrate that the CAM is a high-throughput model for ULM treatment assessment and describe both the qualitative and quantitative observations from the four treatment groups in this study, as compared to gold-standard histology. These observations are then reviewed in the Discussion section.

## II. Materials and Methods

### A. Ethics Approval

Avian embryos, such as the chicken CAMs used in this study, are not considered to be “live vertebrate animals” according to the NIH PHS policy. No IACUC approval was necessary for the chicken embryo experiments presented in this manuscript.

### B. CAM Preparation

Fertilized chicken eggs (white leghorn) were obtained from the University of Illinois Poultry Research Farm and placed into a humidified hatching incubator (Digital Sportsman Cabinet Incubator 1502, GQF manufacturing Inc.). On the fourth embryonic development day (EDD-4), *ex ovo* chorioallantoic membrane (CAM) assays were generated by opening the eggshell with a rotary Dremel tool and transferring egg contents into plastic weigh boats. *Ex ovo* CAMs were housed in a separate humidified incubator (Darwin Chambers HH09-DA) for the duration of the study.

### C. Cell Line Culture and CAM Tumor Engraftment

Human colorectal carcinoma cell lines HCT-116 (ATCC CCL-247) and HT-29 (ATCC HTB-38) were obtained from the American Type Culture Collection Inc. (Bethesda, MD). Both cell lines were maintained in McCoy’s 5A media (Cell Media Facility, UIUC, Urbana, IL, USA) supplemented with 10% fetal bovine serum (FBS, Gibco, Waltham, MA), and 1% pen/strep (Gibco, Waltham, MA). Cells were sub-cultivated when above an 80% confluence level at a 1:4 ratio. Cells were kept in a humidified incubator at 37°C and 5% CO_2_ atmosphere.

The CAM engraftment procedures were modified from the previously reported protocol [32]. Matrigel (BD Bioscience) aliquots were placed in ice water and stored in a 4°C refrigerator to thaw at least two hours prior to tumor engraftment. On the day of tumor engraftment (EDD-09), HCT-116 or HT-29 cells were trypsinized (0.05% Trypsin-EDTA, Gibco, Waltham, MA) for 10 minutes, and detached cells were collected with serum containing media, transferred into 15 mL falcon tubes, and centrifuged for 5 minutes at 300 g. The cell pellets were then re-suspended in PBS, and two 10 μL samples of the cell suspension were transferred into a disposable hemocytometer and analyzed with a Countess cell counter (Life Technologies). Cell containing falcon tubes were then centrifuged again with the same settings as before, the PBS discarded, and the resulting cell pellets were re-suspended in Matrigel to achieve an inoculation dose of 1x10^6^ cells per 10 μL of the cell-Matrigel mixture.

*Ex ovo* chicken embryos were removed from their housing incubator. An autoclaved cotton-tipped applicator was briefly touched to surface of the CAM membrane to expose the superficial vasculature, and 10 μL of the cell-Matrigel mixture was pipetted into the scratch. A total of 52 HCT-116 and 47 HT-29 xenograft bearing CAMs were prepared for this study.

### D. Preparation and Dosing of Reagents

Sorafenib powder (p-Toluenesulfonate salt) was obtained from LC Laboratories (Woburn, MA) and dissolved in dimethyl sulfoxide (DMSO) to generate a stock solution at 200 mg/mL. The stock sorafenib solution was then serially diluted with phosphate buffered saline (PBS) until a final dosing concentration of 30 mg/kg was achieved, assuming a 55 mg egg weight. FOLFOX-6 dosing solution was produced by combining oxaliplatin (Novaplus Vizient Inc.), folinic acid (West-Ward Pharmaceuticals Corp.), and fluorouracil (Fresenius Kabi) to achieve a final dosing solution ratio of 100 mg/m^2^ oxaliplatin, 400 mg/m^2^ folinic acid, and 400 mg/m^2^ fluorouracil. Clinical equivalent doses for the CAM were estimated by assuming an average patient body surface area of 1.79 m^2^ [33] and a body weight of 70 kg. Vehicle control for sorafenib was 5% DMSO in PBS, and vehicle control for FOLFOX-6 was plain PBS. Dosage of sorafenib was performed daily, beginning immediately after the pre-treatment imaging session (EDD-13) and continuing until the study endpoint (EED-17). FOLFOX-6 dosing was performed twice (EDD-14 and EDD-15) for the duration of study. For embryos undergoing combination therapy, a delay of at least 6 hours was used between sorafenib and FOLFOX-6 dosing to mitigate any reaction between the DMSO solvent and the chemotherapy.

Hypoxyprobe powder (pimonidazole hydrochloride, Chemicon) was dissolved in PBS to produce a 116 mg/mL stock solution and was stored at 4°C. Aliquots of the hypoxyprobe stock solution were then further diluted with room temperature PBS to achieve a target dosage of 50 mg/kg pimonidazole on the last day of the study. This hypoxyprobe mixture was intravascularly injected (100 μL) immediately after endpoint ULM imaging, and this was allowed to recirculate for at least 30 minutes before tumor excision.

### E. Ultrasound Imaging

Ultrasound imaging was performed with a Verasonics Vantage 256 programmable ultrasound system (Verasonics Inc., Kirkland, WA) equipped with an L35-16vX linear array transducer (Verasonics Inc.). Plane-wave imaging was performed with a center frequency of 20 MHz, a 1-cycle pulse duration, and a transmit voltage of 2 volts, with 9-angle compounding (−4° to 4°, 1° increment) and a post-compounding effective framerate of 1000 Hz. The maximum depth of imaging was fixed to 2.93 mm, the width of imaging was fixed to 8.82 mm, and the TGC settings were consistent for all imaging acquisitions. The mechanical index (MI) of this imaging acquisition was measured using a 0.075 mm needle hydrophone (SN 3904, Precision Acoustics Ltd., Dorchester, U.K.), yielding a mean MI value of 0.015. A fresh glass needle was prepared for contrast agent injection by pulling a glass capillary tube (B120-69-10, Sutter Intruments, Novato, CA, USA) with a PC-100 glass puller (Narishige, Setagaya City, Japan). The glass needle was then secured into 8 cm of Tygon R-3603 laboratory tubing, and the other open end of the tubing was fitted over a 18Gx1.5 BD PrecisionGlide needle with 1 mL syringe. A single 70 μL bolus of a clinically used ultrasound contrast agent (Definity, Lantheus Medical Imaging, N. Billerica, MA, USA) was manually injected into a superficial CAM surface vessel (~50 μm diameter) with the aid of a Nikon SMZ800 stereomicroscope (Nikon, Tokyo, Japan). The injection bolus was pushed slowly to avoid microbubble destruction from the small glass needle opening, requiring approximately one minute for the full 70 μL volume. Although, clinically, a microbubble bolus is followed by a saline flush, this is not necessary for CAM injections as the micro-perfusion patency can be readily visualized to confirm the venous return of the microbubbles. The chicken embryo was then immediately moved to the adjacent ultrasound imaging system for ULM acquisition, where the transducer was placed to produce the largest imaging cross-section of the tumor. It has been demonstrated that microbubbles will remain in circulation for several hours in the chicken embryo [34], so although the time delay between the microscope-guided microbubble injection and ultrasound imaging precludes conventional contrast-ultrasound indicator-dilution quantifications, there will remain sufficient recirculating microbubbles for ULM reconstruction. Each CAM tumor was imaged using 20 separate acquisitions of 1600 frames each, resulting in a total acquisition length of 32000 frames (32 seconds) per imaging session. We conducted serial imaging of each CAM tumor including both a baseline scan (EDD-13; prior to treatment) and an endpoint scan (EDD-17). The placement and alignment of the transducer was marked on the side of the chicken embryo-housing weigh boat during baseline imaging to ensure that the subsequent imaging session maintained an equivalent tumor orientation. Ultrasound imaging acquisitions were stored as in-phase/quadrature (IQ) demodulated data for ULM processing in MATLAB.

### F. Contrast-Enhanced Ultrasound Signal Processing

Background tissue signals were suppressed by applying a spatiotemporal SVD-based clutter filter to the contrast-enhanced IQ data [28], [35-37]. Each 1600 frame IQ dataset was reshaped into a 2D Casorati matrix, an SVD decomposition was then performed to separate out the singular values of the data, and the low-order values were zeroed out to remove tissue signal. The cutoff threshold was determined adaptively as in Song *et al*. [38], and typically filtered out the first 10-20 singular values. The filtered data was then recovered via inverse SVD and was reshaped back into a 1600 frame IQ data size. A noise-equalization profile was generated by applying the same global SVD filtering to the noise reference data [39]. This was then applied to the SVD-filtered MB data to equalize the MB intensity through-out the imaging field of view. Diffraction-limited contrast enhanced power images were generated by accumulating the MB signal power for each IQ data over time.

### G. Super-Resolution Image Reconstruction

The SVD-filtered, noise-equalized IQ data was spatially interpolated to a 5 μm axial/lateral resolution using a 2D spline interpolation. A point-spread function (PSF) representing an individual MB was generated using a multivariate Gaussian function, with the axial and lateral dimensions adjusted based on the imaging dataset. We then applied a 3D conical Fourier domain filter to split the IQ dataset into three different subsets in an attempt to separate overlapping MB populations [36]. For each of these subsets, a normalized 2D cross-correlation was performed to localize MBs on every frame. We then applied a threshold to reject pixels with a low cross-correlation coefficient [28], [36], [40] and localized cross-correlation peaks with the “imregionalmax.m” function in MATLAB, and stored the resulting MB centroids. Pairing and tracking of MB centroids was performed using the uTrack algorithm [41] in MATLAB, with a minimum pairing persistence of 10 frames.

### H. Ultrasound Image Analysis

Manual segmentation of the tumor cross-sectional area region of interest (ROI) was performed in MATLAB using Bezier control points and an interpolating spline using Hobby’s algorithm [42]. The tumor maximum diameter was calculated by finding the longest distance between two boundary points of the ROI, analogous to the RECIST long-axis diameter. Tumor contrast power was calculated by accumulating the MB signal for the diffraction-limited contrast images within the ROI along the temporal dimension. Tumor blood volume of ULM images was estimated by accumulating the total number of MB centroids within the ROI. Intervessel distance was calculated by binarizing MB centroid maps to identify avascular regions, and then determining the distance to the closest vascularized pixel. The DM and sum-of-angles (SOAM) metrics were calculated as described by Shelton *et al*. [43]. Finally, the Hausdorff fractal dimension of the tumor vasculature was estimated using a box-counting algorithm on the ULM vascular maps [44].

### I. Histology and Immunohistochemistry

Following ultrasound imaging, CAMs were intravascularly injected with the hypoxyprobe solution as detailed above. After ample circulation time, tumors were excised using forceps and dissection scissors and were placed into a container with 10% neutral buffered formalin (NBF) for fixation at room temperature. After 24 hours of fixation, the NBF solution was replaced with 70% ethanol for storage. Tumors were submitted to the University of Illinois at Urbana-Champaign Veterinary Diagnostic Laboratory for paraffin embedding, sectioning, and immunohistochemistry. Fixed tumor tissue was sectioned at 4 μm, and unstained sections were blocked at room temperature with 2% bovine serum albumin (VWR, Batavia, IL, USA) and 10% fetal bovine serum (VWR, Batavia, IL, USA) for one hour. Unstained sections were incubated using a Hypoxyprobe™ plus kit (Hypoxyprobe, Inc., Burlington, MA, USA) which includes a primary antibody anti-pimonidazole (0.6 uL/mL mouse IgG1 monoclonal antibody 4.3.11.3, Hypoxyprobe, Inc., Burlington, MA, USA) and a secondary reagent (HRP conjugated rabbit anti-FITC, Hypoxyprobe, Inc., Burlington, MA, USA). These were then stained with DAB chromogen (33′-diaminobenzidine tetrahydrochloride, Pierce Biotechnology Inc., Waltham, MA, USA) to identify hypoxyprobe accumulation. Another set of unstained sections were incubated with Ki67 monoclonal antibody (Agilent, Santa Clara, CA, USA) and stained with DAB chromogen to identify actively proliferating cancer cells.

### J. Histological Quantification

Histological slides were imaged with a NanoZoomer HT slide scanner (Hamamatsu, Hamamatsu City, Japan) at a 20x objective and saved as .ndpi files. Digitized slide sections were imported into MATLAB and the DAB staining for either hypoxyprobe or Ki67 was extracted using the technique proposed by Pham *et al*. [45]. Tumor cross-sections were manually segmented using Hobby’s algorithm [42] and the H-score for each tumor was calculated [46].

### K. Statistics

Statistical analysis was performed in the R programming language [47]. Boxplots were generated using the ggplot2 package [48]. For histological quantifications (only end-point data available) we used a one-way analysis of variance (ANOVA) to test statistical significance and applied a Tukey’s honestly significant difference test to correct for multiple comparisons. For longitudinal comparisons we used an analysis of covariance (ANCOVA) on the post-treatment scores with the pre-treatment value as a covariate and the treatment as an independent variable. In all cases a p < 0.05 was considered to be statistically significant.

## III. Results

### A. The CAM Tumor Xenograft Model Permits High-Throughput ULM Treatment Screening

**Fig. 1.**
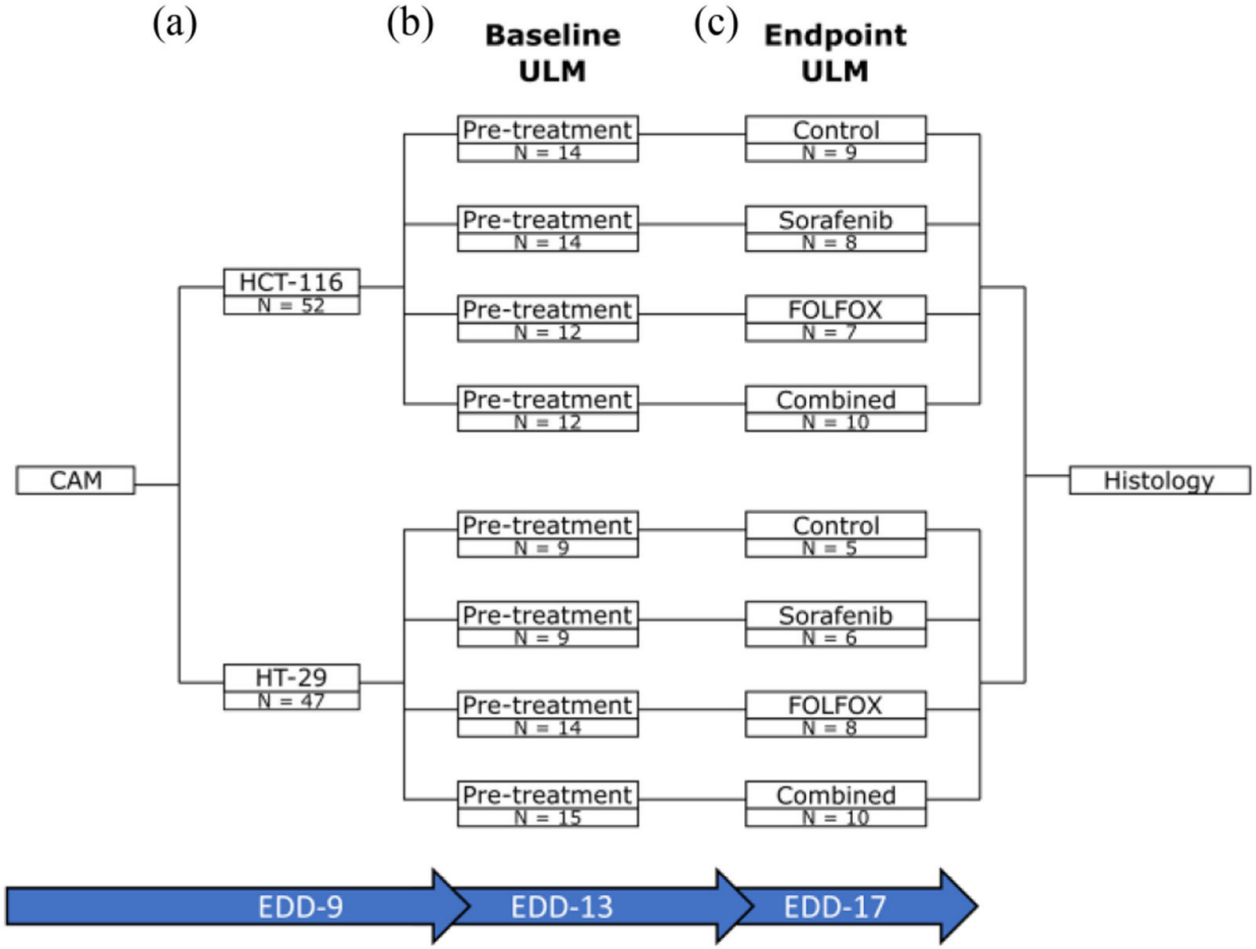
Study design diagram. A) At EDD-09 a total of 52 CAMs were engrafted with the HCT-116 cell line and 47 CAMs were engrafted with the HT-29 cell line. B) The tumor engrafted CAMs were randomized, and a pretreatment ULM imaging session was performed on EDD-13. C) The tumors underwent their selected therapy as described in the methods section, and a post-treatment ULM imaging session was performed on EDD-17. Tumors were excised immediately after imaging and formalin fixed for histology.

We have previously reported that the CAM of chicken embryos provides a highly accessible and easy-to-implement tumor xenograft model for cancer therapy evaluation [32] and ULM imaging [28]. This permitted a high-throughput ULM imaging platform for treatment screening, with a total of 52 HCT-116 tumors and 47 HT-29 tumors for the baseline (pre-treatment) imaging session on EDD-13 (Fig. 1(A-B)). Although there was some chicken embryo attrition noted in all of the randomized treatment groups (Fig. 1(C)), this study still had 34 HCT-116 tumors and 29 HT-29 tumors for the endpoint (post-treatment) imaging session on EDD-17, with at least N ≥ 5 in each treatment group. This level of attrition is not unexpected for the *ex ovo* chicken embryo model, which routinely sees attrition rates approaching 50% [34], but the low relative cost of chicken embryo xenograft models allows for a large safety margin in study planning to ensure that there will be sufficient samples at the endpoint.

### B. Super-Resolution ULM Imaging Reveals Exquisite Microvascular Density Maps of CRC CAM Tumors

**Fig. 2.**
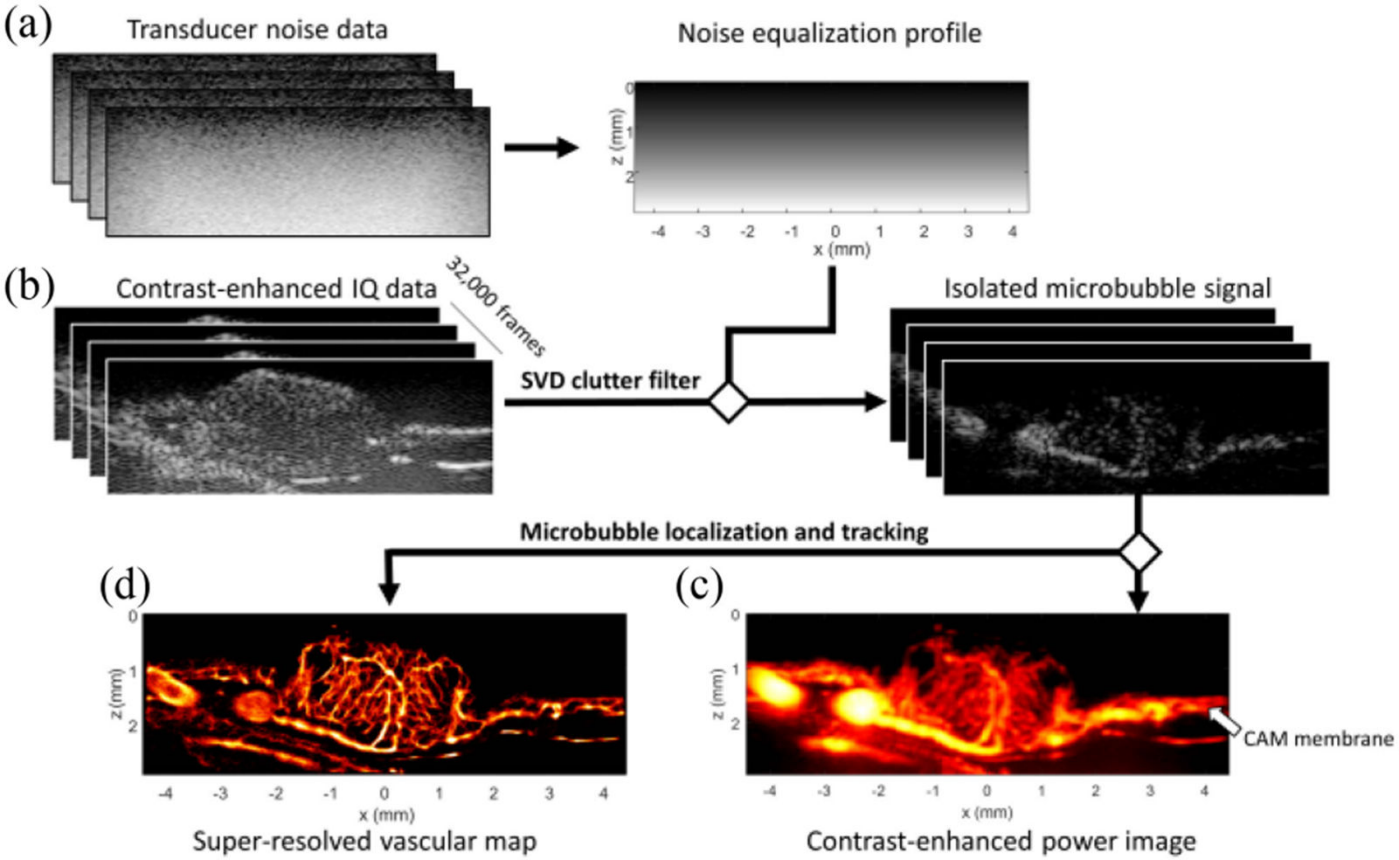
ULM acquisition and processing pipeline. A) A reference transducer acquisition was used to estimate a noise equalization profile to correct for depth dependent attenuation. B) A total of 32000 contrast-enhanced IQ data frames were acquired and SVD filtered to generate isolated microbubble data and C) contrast-enhanced power Doppler images. The isolated microbubble data then went into the ULM processing pipeline, which included microbubble separation, microbubble localization, and microbubble pairing and tracking. D) The final super-resolved images were used to quantify the treatment effect in each CAM tumor therapy group.

As ultrafast planewave imaging does not have transmit focusing, it was necessary to estimate a noise equalization profile for the L35-16vX transducer to compensate for the depth-dependent ultrasound system noise (Fig. 2(A)). Isolated MB flow data was extracted following SVD clutter filtering and noise equalization (Fig. 2(B)), and the clinical concentration MB bolus was divided into sparser subsets of imaging data using a MB separation algorithm [36] to improve localization and tracking fidelity. By accumulating the MB signal over the imaging ensemble, a contrast-enhanced power Doppler image could be generated for this tumor dataset (Fig. 2(C)), yielding a blood volume map demonstrating predominately centralized vasculature. The highly vascularized planar tissue adjacent to the tumor mass is the CAM membrane (Fig. 2(C), white arrow), which provides all vascular supply to the tumor. Super-resolution ULM images (Fig. 2(D)) from these tumors reveal a chaotic vascular appearance with reduced peripheral vascularization and some large feeding vasculature apparent in the tumor tissue closest to the membrane surface.

### C. Longitudinal ULM Imaging Confirms the Gradual Development of Intratumoral Vasculature

**Fig. 3.**
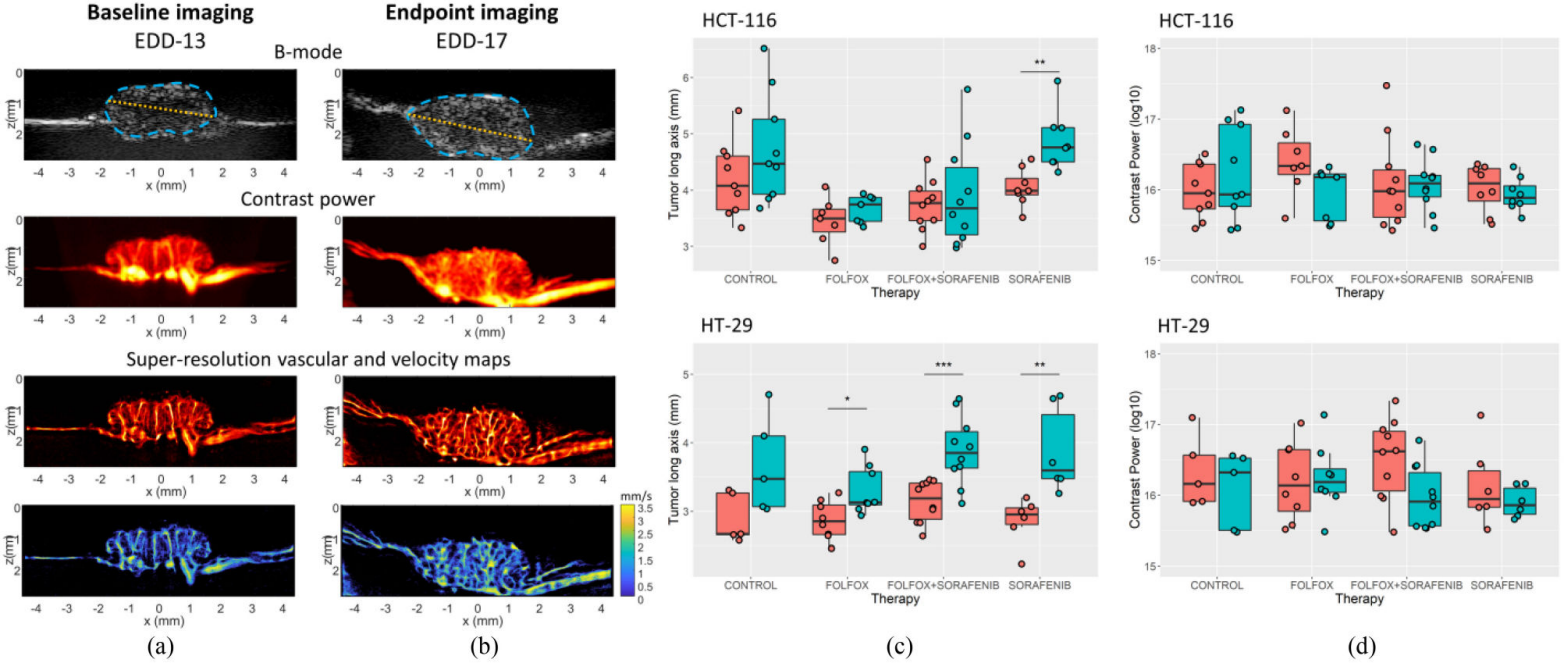
Longitudinal changes in CAM tumors. A) Representative baseline contrast-enhanced power Doppler and super-resolution ULM images of CAM engrafted tumors. B) This same control tumor at endpoint imaging, which demonstrates both the growth of the tumor diameter and the continued development of intratumor vascularization over the course of the study. Tumor outlines and maximal diameter are demonstrated as cyan and orange lines on the B-mode image, respectively. C) Quantification of the tumor long-axis which reveals a general trend of tumor growth for all treatment groups in this study. D) Quantification of the contrast power of the tumor cross-sectional area.

Baseline (pre-treatment) imaging of the CRC CAM tumors (Fig. 3(A)) reveal a relatively small tumor mass with sparse, columnar microvasculature originating from the CAM vascular bed. An example tumor outline and maximal length are demonstrated on the B-mode images as cyan and orange lines, respectively. At the study endpoint, tumor cross-sections were larger and had a more developed intratumoral vascular network (Fig. 3(B)). Maximal tumor length generally increased in all treatment groups for the two cell lines examined in this study (Fig. 3(C)). Treatment with sorafenib demonstrated a large, significant increase in the tumor long axis for both the HCT-116 and HT-29 cell lines. This is consistent with our previous observations of AA-treated renal cell carcinoma tumors on the chicken embryo [32]. For the HCT-116 cell line, the only significant increase in the tumor long axis was for the sorafenib treated group (*p* = 0.002). For HT-29 tumors, we found a significant increase for the FOLFOX treated cohort (p = 0.016), the sorafenib treated group (p = 0.006), and the combination therapy (p < 0.001). The HT-29 control group did not reach significance (p = 0.061), likely due to the small remaining N number in this cohort (N = 5 tumors at endpoint). The quantification of contrast power (Fig. 3(D)) demonstrated a trend toward reduced contrast enhancement in treated tumors, but no significant difference was found between treatment groups.

### D. Tumor Therapy Condition Yielded Differences in ULM Vascular Characteristics

**Fig. 4.**
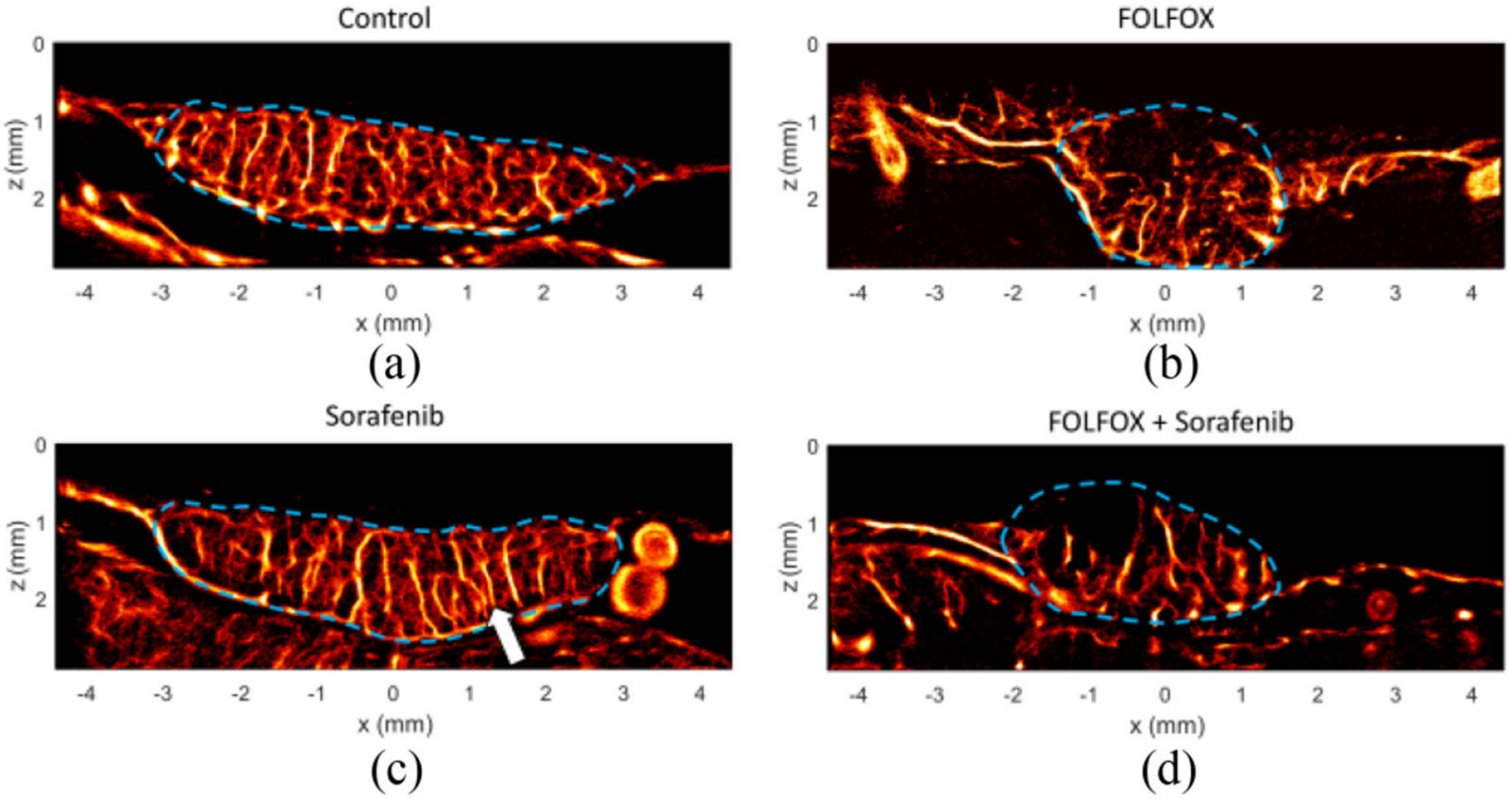
Super-resolution images of treatment effect. A) Endpoint (EDD-17) control ULM images reveal a dense microvascular network throughout the tumor mass. B) In comparison to the control tumors, the sorafenib treated tumors exhibited slight vascular pruning of microvessels, with a more columnar appearance in vascular network structure (arrow). C) Chemotherapy treated tumors displayed avascular tumor regions, implying a more aggressive vascular pruning. D) Combination treated tumors also exhibit avascular tumor regions.

Control treated tumors (Fig. 4(A)) possessed a chaotic microvascular appearance with little obvious directionality in the formation of blood vessels. In comparison, sorafenib treated tumors exhibited evidence of slight vascular pruning of microvessels and had a more columnar appearance (arrow) in the larger prominent vessels passing through the tumor from the CAM vessel bed (Fig. 4(B)). The two treatment groups that included chemotherapy (FOLFOX-6 and the combined therapy group) displayed avascular tumor regions, particularly in regions distant from the CAM membrane (Fig. 4(C-D)). This qualitative observation implies that the two treatment regimens that include chemotherapy had a more pronounced vascular disruption in this tumor model than in the AA monotherapy group.

### E. Super-Resolution Vascular Maps are Qualitatively Similar to Histology

**Fig. 5.**
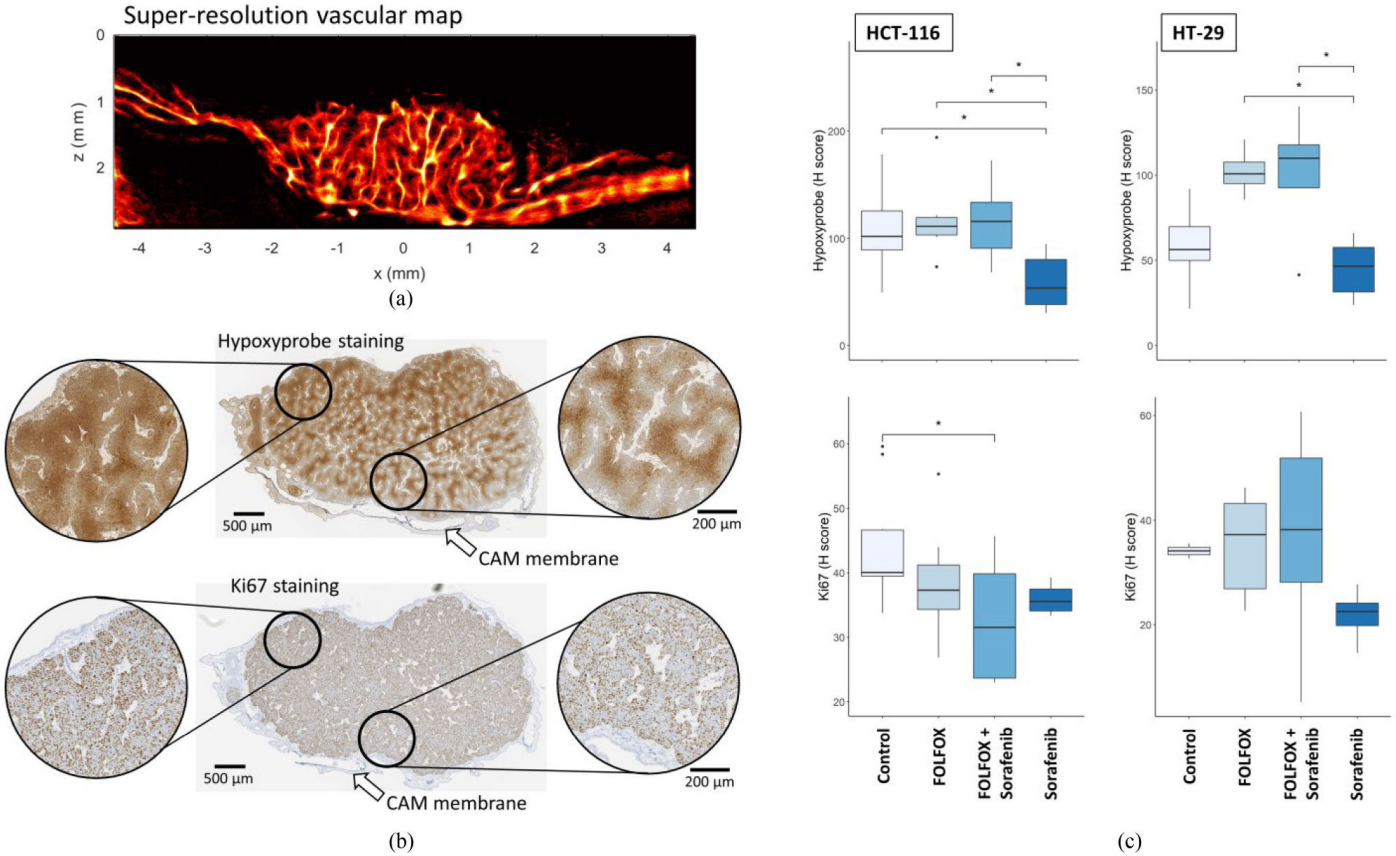
Histological comparison and quantifications. A) The super-resolution vascular map can be compared qualitatively to the B) histological staining in cases where the imaging plane was relatively aligned to the histology cross-section. Hypoxyprobe IHC, a marker of hypoxia, demonstrated darker staining in the tumor regions distant from the CAM membrane (left inset). In comparison, the regions closer to the membrane had reduced hypoxia staining (right inset). Ki-67 staining was prevalent across the depth of the tumor. C) Quantifications of the hypoxyprobe staining (top row) and Ki67 staining (bottom row) for both the HCT-116 and HT-29 cell lines for all treatment groups.

Although it was not explicitly designed into the study, in some instances the ultrasound imaging plane was partially aligned with the histological cross-section (Fig 5(A) and (B)), which allows for qualitative comparisons between the ULM imaging reconstruction and IHC measures of tissue hypoxia and cell proliferation. Hypoxyprobe staining (Fig. 5(B)), a marker of intratumoral hypoxia, demonstrated a mottled staining pattern throughout the tumor mass, implying the presence of an intratumoral vascular network. In the tumor regions distant from the CAM membrane (left inset) the hypoxyprobe staining was darker and more uniform, indicating that these regions were experiencing more hypoxic stress. Closer to the CAM (right inset), the level of hypoxia was reduced, and the staining pattern was more varied. Ki-67 staining, a marker of cellular proliferation, demonstrated a punctate staining appearance in the tumor tissue, with strong staining both in the tumor area distant from the CAM (left inset) and in regions closer to the CAM membrane (right inset). Quantifications of the histological staining (Fig. 5(C)) demonstrate significantly reduced hypoxyprobe staining in the sorafenib treated tumors. For HCT-116 tumors, the hypoxyprobe H-score was significantly different between control and sorafenib (p = 0.035), FOLFOX-6 and sorafenib (p = 0.023), and the combination and sorafenib (p = 0.015). For the HT-29 cell line, both chemotherapy treated groups demonstrated a trend of increased hypoxia in comparison to control, and were significantly higher than the sorafenib monotherapy (FOLFOX-6 vs. sorafenib p = 0.018, combination vs. sorafenib p = 0.022). For cell proliferation staining, the HCT-116 cell line demonstrated a trend toward reduced Ki-67 staining for all treatment groups in comparison to the control group, with a significant difference detected for the combination therapy treated group (p = 0.016). No significant effect was found for Ki-67 staining in the HT-29 cell line.

### F. Super-Resolution Quantifications of Longitudinal ULM Imaging

**Fig. 6.**
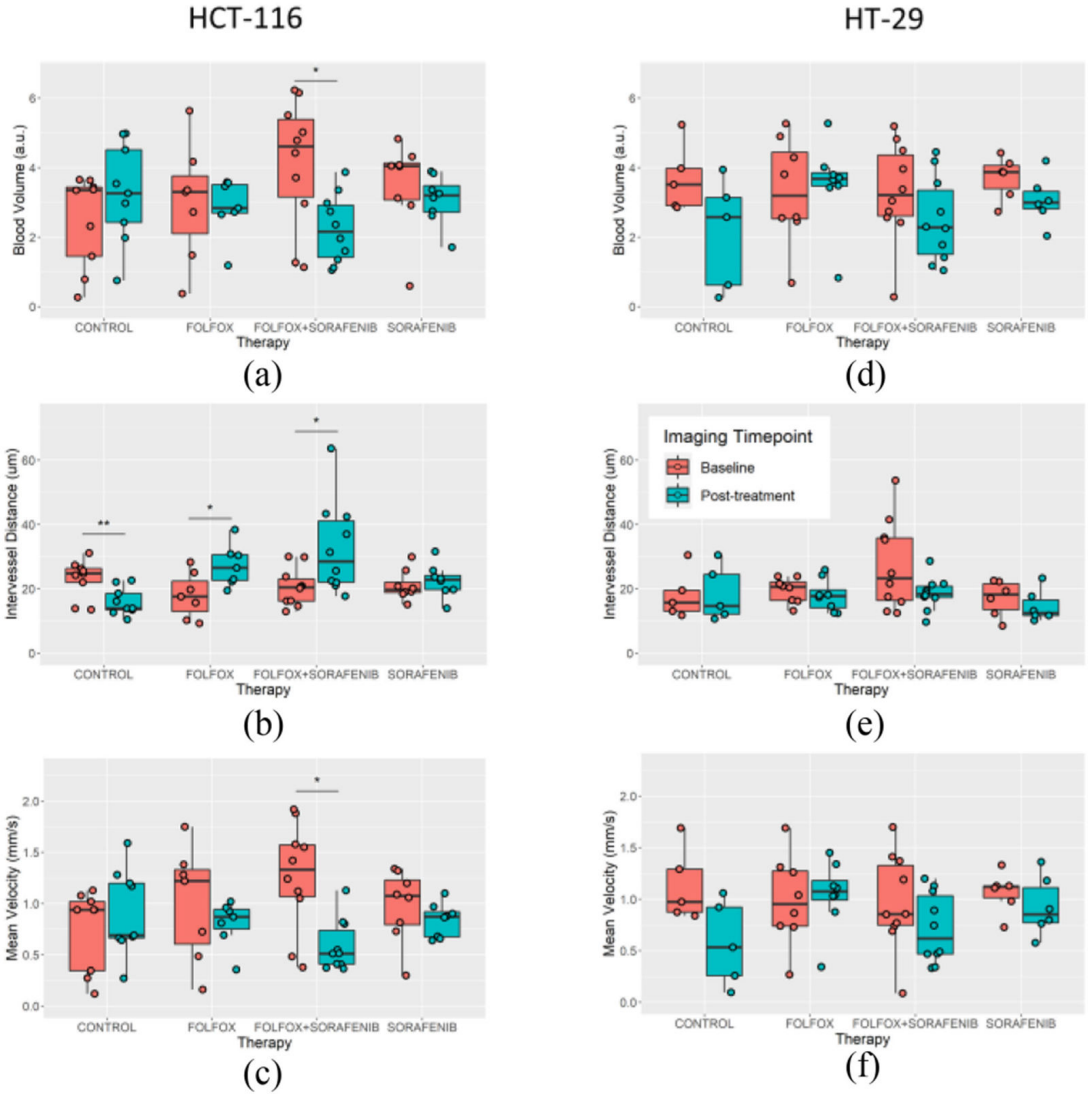
Quantification of tumor vascularization. A) HCT-116 tumors showed a trend toward reduced blood volume for the cohorts treated with sorafenib, with a significant difference detected in the combination therapy group. B) The intervessel distance for this cell line demonstrated significant decreases for the control group, increased for both chemotherapy treatments, and little change for the sorafenib monotherapy. C) Tumor mean blood velocity trended toward slower flow, in particular for the combination therapy group. D) HT-29 tumors also demonstrated a longitudinal trend of reduced blood volume, but no significance difference was detected. E) Intervessel distance was relatively static for the HT-29 cell line for all treatment groups. F) Mean blood velocity also demonstrated a trend of reduced velocity, but no significance was found.

Super-resolution ULM imaging of the CAM engrafted tumors permitted quantification of the tumor microvascular features and metrics of vascular tortuosity. For the HCT-116 cell line, there was a trend toward reduced blood volume for both therapy groups that included the AA sorafenib (sorafenib monotherapy and the combination therapy, Fig. 6(A)). A significant difference was detected for the combination therapy group (p = 0.01). The intervessel distance (Fig. 6(B)) for this cell line demonstrated a significant decrease for the control group (p = 0.009), indicating that the tumors became more densely vascularized over the course of the study. In comparison, the FOLFOX-6 and combination groups showed an increase in intervessel distance (p = 0.025 and p = 0.023, respectively) after therapy, potentially due to vascular disruption by chemotherapy. The sorafenib treated HCT-116 tumors had a relatively consistent intervessel distance before and after therapy. Tumor blood velocity demonstrated a trend toward slower flow (Fig. 6(C)), with significance found in the combination therapy group (p = 0.002). The HT-29 tumors exhibited a longitudinal trend of reduced blood volume (Fig. 6(D)) for all treatment groups, but no significant differences were detected. Likewise, intervessel distance was relatively static for the HT-29 tumors (Fig. 6(E)) regardless of the therapy, and no significance was detected for mean blood flow velocity (Fig. 6(F)).

**Fig. 7.**
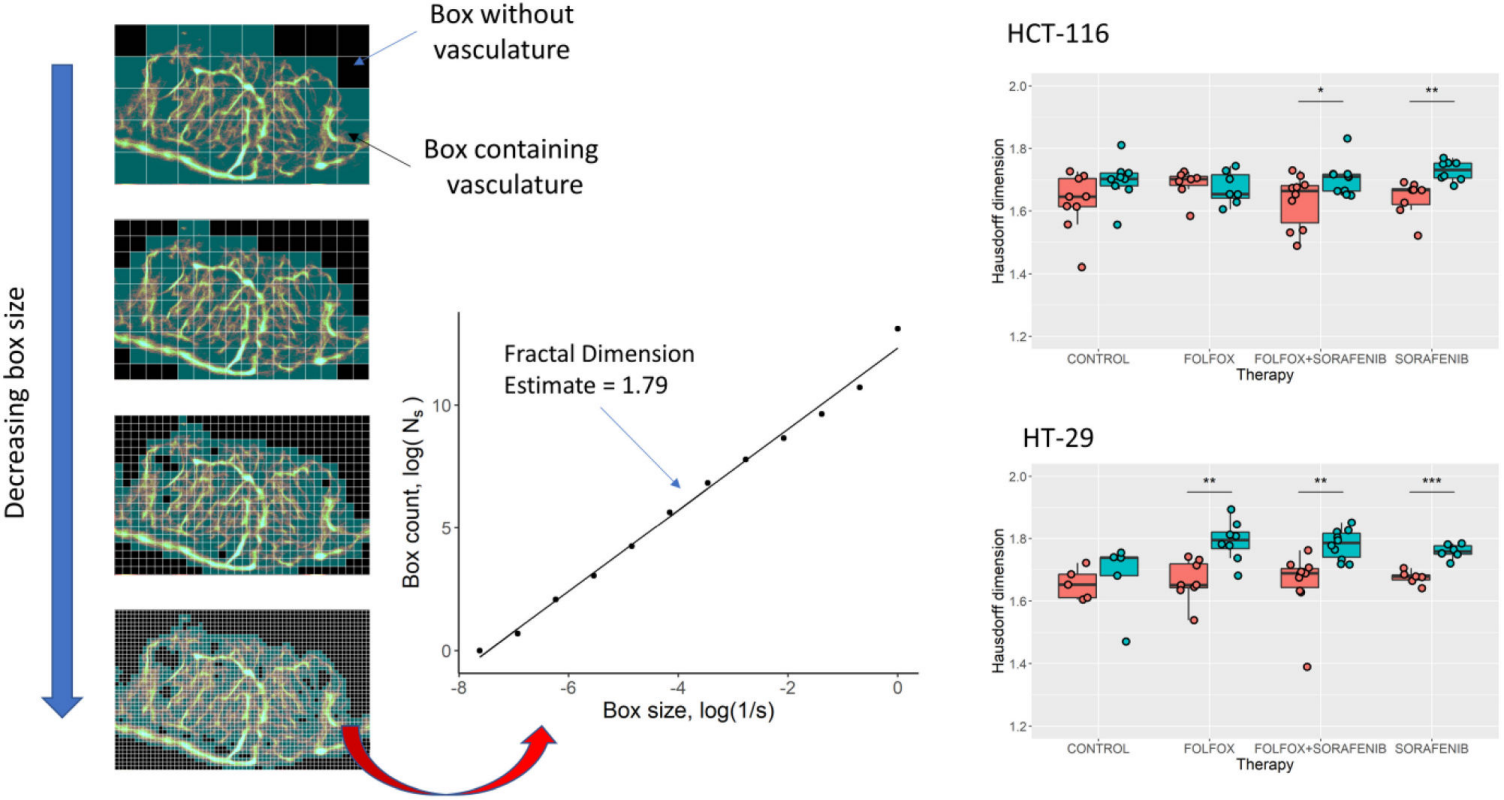
Hausdorff box-counting of ULM images. The Hausdorff fractal dimension of ULM vascular maps was estimated using the slope generated using a multi-dimensional box-counting algorithm. Interestingly, all tumor groups treated with the AA agent sorafenib showed a significant increase in Hausdorff dimension. The HT-29 FOLFOX-treated tumors also demonstrated a significant increase in Hausdorff dimension but the opposite trend was noted in HCT-116 tumors treated with FOLFOX.

**Fig. 8.**
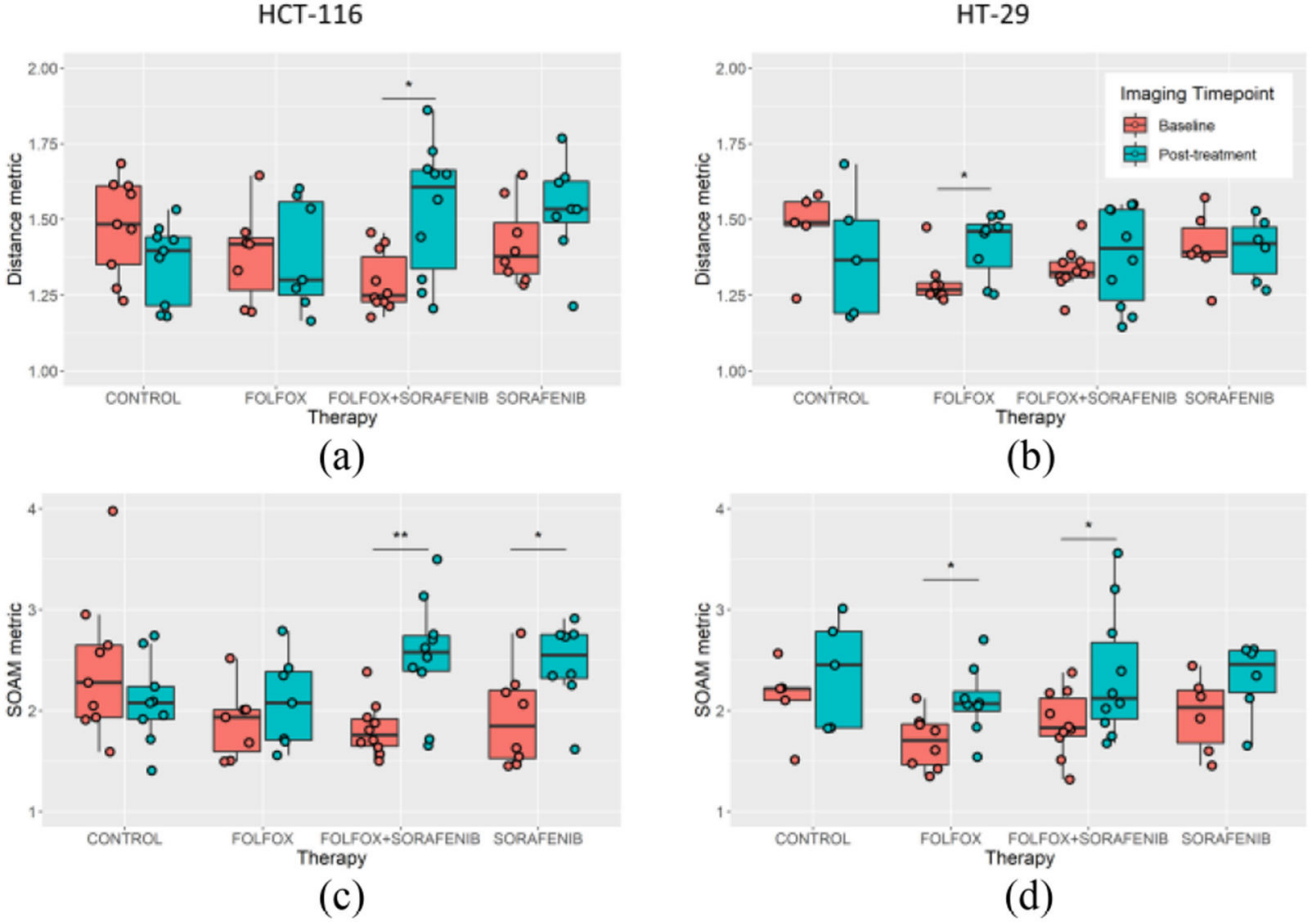
Measures of vascular tortuosity. Vascular tortuosity was measured using the distance metric (DM) and the sum of angle metric (SOAM). For the HCT-116 cell line, the A) DM was found to significantly increase in the combination therapy group and B) SOAM measures were found to significantly increase in both sorafenib containing therapies. The HT-29 cell line showed C) a significantly increased DM for FOLFOX treated tumors, and D) increased SOAM in the FOLFOX and combination therapy treated groups.

The vascular tortuosity of the pre- and post-treatment tumors was measured on the ULM images using three established metrics: the distance metric (DM), the sum of angles metric (SOAM), and the Hausdorff fractal dimension (Fig. 7). A significant difference was detected in the Hausdorff dimension of the sorafenib monotherapy and combination therapy groups for both tumor cell lines (p = 0.002 and p = 0.034 for HCT-116, and p < 0.001 and p = 0.003 for HT-29, respectively). The FOLFOX-treated HT-29 tumors also demonstrated a significant increase in Hausdorff dimension (p = 0.002). Based on the DM (Fig. 8) there was trend toward decreased tortuosity in control HCT-116 and HT-29 treated tumors, and a trend toward increased DM in the therapy groups. A significant increase in DM was found for combination therapy-treated HCT-116 tumors (Fig. 8(A), p = 0.006) and for FOLFOX treated HT-29 tumors (Fig. 8(C), p = 0.022). For the SOAM, a significant increase was found in the HCT-116 tumor groups (Fig. 8(B)) treated with either AA monotherapy (p = 0.029) or the combination therapy (p = 0.002). The HT-29 cell line tumors demonstrated a significant increase for the SOAM (Fig. 8(D)) for the FOLFOX (p = 0.020) and the combination therapy (p = 0.048).

## IV. Discussion

This study examined the utility of super-resolution ULM imaging to quantify the treatment effects of sorafenib monotherapy, FOLFOX-6 therapy, and a combined chemotherapy/AA regimen on two human CRC tumor xenograft models (HCT-116 and HT-29 cell lines) in the chicken embryo and compared the results with histological quantifications of tumor hypoxia and proliferative potential. We demonstrated that the chicken embryo xenograft model is an inexpensive and high-throughput animal model for ULM imaging, permitting large N number therapy-control studies of vascular effects, and that high-fidelity vascular images can be reconstructed in the context of a longitudinal therapy screening study. To our knowledge, this is the first study demonstrating the application of longitudinal ULM to a large number (N = 63) of tumors undergoing anti-cancer and/or AA treatments, and the first study to apply fractal-based characterization estimates to ULM vascular reconstructions in tumors. This is also the first study attempting to characterize synergistic treatment effects from combination therapies using ULM imaging.

We found that the HCT-116 cell line xenografts were more sensitive to applied therapies than the HT-29 cell line xenografts. This is consistent with reports in literature, which have demonstrated that HCT-116 cells *in vitro* are more apoptotic than HT-29 cells when exposed to FOLFOX therapy [49], and that HCT-116 cells exhibit less colony density and a reduced xenograft size in mice in comparison to HT-29 xenografts when exposed to sorafenib therapy [50]. This observation could be explained by the different expression and secretion of vascular endothelial growth factor by these two cell lines and the role of sorafenib in disrupting the autocrine signaling that promotes cell proliferation [51], along with the differential response of these two cell lines to hypoxic stress [52]. At the clinically relevant doses used in this study, the HT-29 tumors grew under all therapies in comparison to only the sorafenib treated group for HCT-116 (Fig. 3). Although the increase in tumor size for the sorafenib treated tumors is surprising, it is consistent with our previous work on AA treated renal cell carcinoma tumors on the chicken embryo [32]. This could indicate a pseudo-progression phenomena [53], given the short time frame of this study, or may be an inflammatory effect inherent to the chicken embryo tumor xenograft model. It should also be noted that in our previous study, there was a difference in the amount of vascular reduction depending on the AA agent used (sunitinib or pazopanib), indicating that similar clinical equivalent doses of different targeted therapies may have distinct phenotypic and/or functional effects in the CAM tumor model. This likely depends on the nature of the targeted therapy and on the molecular characteristics of the specific cancer cell line engrafted into the CAM. The HT-29 tumors also did not demonstrate a significant effect on their vascularization, whereas the HCT-116 tumors exhibited vascular pruning and/or vascular disruption, which was the most pronounced for the combination therapy group (Fig. 6). Although the direct translatability of CAM tumor xenograft responses to clinical outcomes is unknown, there is some evidence in literature that the CAM tumor model can be used to screen for treatment efficacy. For example, Marimpietri *et al*. [54] evaluated the synergistic effects of a combination of vinblastine and rapamycin, which function in part through anti-angiogenic and cyto-reductive mechanisms, on patient-derived human neuroblastoma xenografts implanted into the CAM. This group was able to replicate this synergistic effect in a mouse orthotopic xenograft model [55], and a Phase I clinical trial [56] demonstrated a clinical response in pediatric patients undergoing this combination therapy.

Histological quantification confirmed that the HCT-116 cell line was experiencing reduced proliferative potential, particularly for the combination therapy cohort, whereas no significant interaction was found for the HT-29 xenografts (Fig. 5). An interesting observation is that the sorafenib treated tumors had the lowest level of intratumoral hypoxia (Fig. 5) but did not show any evidence of a change in intervessel distance (Fig. 6). This, in combination with the observation that the sorafenib treated tumors exhibited a more columnar vessel appearance (Fig. 4(B)), may indicate that there was a functional improvement in the tumor microvasculature which increased oxygenated blood delivery into the xenograft. Given the short time frame of this study, a possible interpretation is that this effect is a transient vascular normalization phenomena [7] that has the potential to increase tumor drug uptake and relieve a hypoxic tumor microenvironment, which is known to promote chemotherapy resistance. The characterization of the tumor vascular fractal dimension (Fig. 7) and tortuosity (Fig. 8) mirrors this interpretation. We found that post-treatment HT-29 tumors demonstrated increasing Hausdorff fractal dimension, potentially indicating a responsive heterogeneity in local pro-angiogenic substrates. We also found increasing DM and SOAM metrics for HCT-116 tumors treated with the AA agent, or the combination therapy, but not for chemotherapy alone. However, these results must be understood within the limitations of the CAM tumor xenograft model, which exhibits a high degree of attrition (Fig. 1(C)) and therefore may introduce a tumor survival bias when interpretating longitudinal results. Future work is required to determine which quantitative indices can detect these physiological states with the goal of leveraging any synergistic interaction with cyto-reductive therapies and for screening of non-responsive patients. Patients exhibiting early radiological indications of poor vascular treatment response (e.g., increasing fractal dimension) may be better served by switching from this non-effective front-line therapy to a second-line therapy wherein the treatment effect could be reassessed. If successful, this would position ULM as a clinically translatable imaging modality that could permit individualized treatment strategies based on microvascular structure and function.

It is important, though, to mention the pragmatic challenge of the clinical translation of ULM given the long imaging acquisition times in this study. Each 1600-frame IQ dataset took about 10 seconds to acquire, which includes data acquisition, data transfer, beamforming, and data storage. Thus, the total imaging time required for each tumor in this study was around 200 seconds, which is beyond a reasonable breath-hold duration. There is also a substantial computational burden for ULM: the total reconstruction time for each tumor was roughly 320 minutes on our desktop workstation (20 Core Intel(R) Xeon(R) Gold 6138 CPU @ 2.00GHz, 1995 MHz, with 128 GB DIMM RAM @ 2666 MHz, and an NVIDIA Quadro P4000). With that said, pilot clinical studies have demonstrated the feasibility of generating super-resolution ULM images of healthy and diseased organs, including tumors, under a single breath-hold from patients [57], and novel ULM reconstruction paradigms have substantially reduced processing times [21].

Another limiting factor for the use of the CAM tumor xenograft model is the short timeframe of tumor viability. For the methodology used in this manuscript, the tumors were only implanted for a total of 8 days (EDD-09 to EDD-17) and only experienced treatment for 4 days (EDD-13 to EDD-17). The treatment effect for clinical equivalent therapy doses is therefore expected to be subtle in this framework, as evidenced by the lack of a clear objective response in chemotherapy treated xenografts. Increasing the drug dosage above clinical recommendations would allow for a more distinct treatment effect but would limit the translatability of the study results. However, the low cost and high N number afforded by ULM imaging of CAM tumor xenografts allows for dose-escalation studies to examine vascular mechanisms of therapy resistance. A longer duration longitudinal screening study in other preclinical xenograft models (e.g., murine models of CRC) would permit a more direct testing of ULM’s ability to stratify tumor responsiveness to chemotherapy based on ultrasound imaging biomarkers. Finally, the results presented in this manuscript must be understood within the context of CAM ultrasound imaging, which represents a near ideal scenario for ULM reconstruction: there is minimal tissue motion, limited attenuation, and shallow imaging depth. These advantages potentially limit the direct translatability of the study’s analysis metrics without modification to other animal models and clinical use due to the impact of attenuation, phase aberration, and motion on MB signal-to-noise ratio and ULM performance. ULM has been successfully applied to numerous pre-clinical and clinical studies, and the results presented in this manuscript serve to motivate additional investigation into ULM radiological features and quantification strategies.

## V. Conclusion

ULM imaging, both in this study and in literature, has demonstrated a substantial improvement in vascular imaging fidelity over conventional Doppler imaging. By overcoming the diffraction limit through the tracking of intravascular MBs, ULM provides additional utility for tumor therapy response evaluation by offering a myriad of morphological and functional quantitative indices for gauging treatment effect. At an imaging resolution approaching the capillary scale, the examination of microvascular structure can be informative of the tumor microenvironment, such as quantifying intervessel distance and vascular tortuosity, which can be critical for detecting early treatment effects. In this study, chemotherapy response was associated with increased intervessel distance, implying a vascular pruning effect, whereas resistance to therapy was associated with increasing Hausdorff fractal dimension as measured by a box-counting technique. The clinical utility of super-resolution ULM radiological features is an ongoing and rapidly developing area of research.
